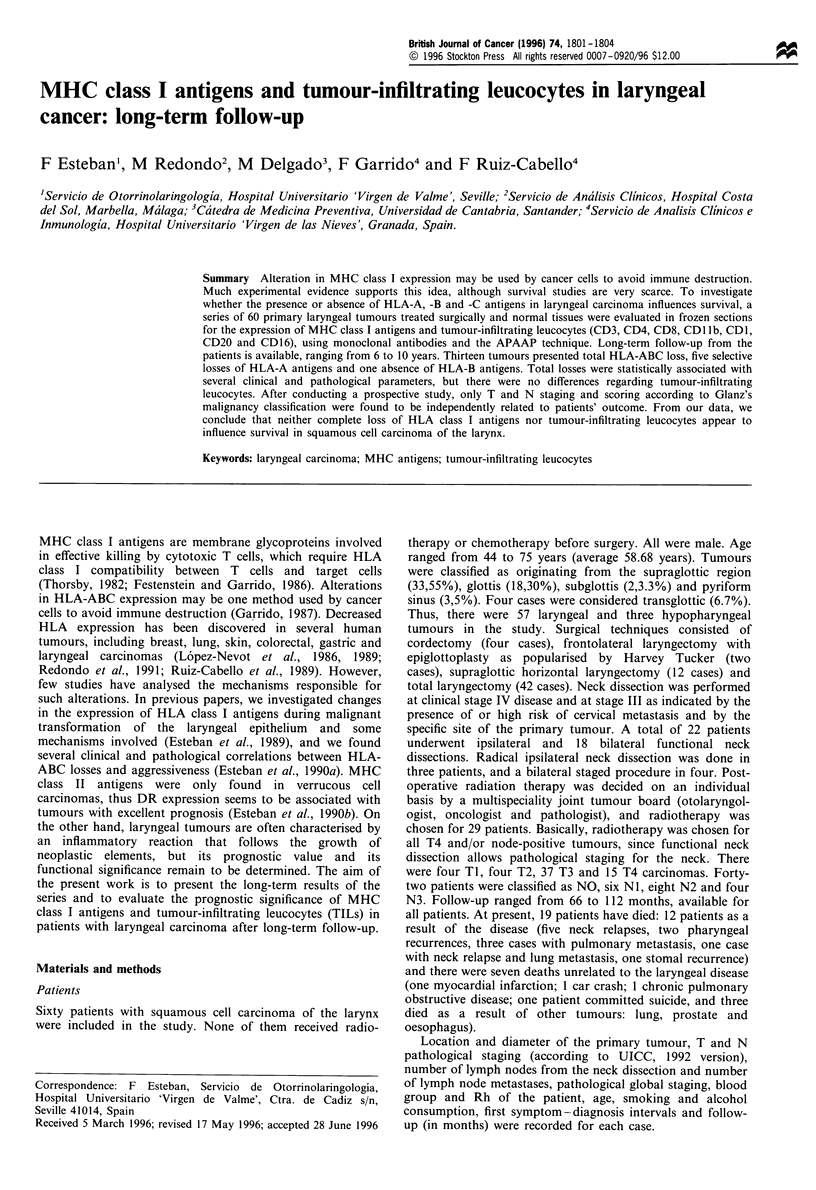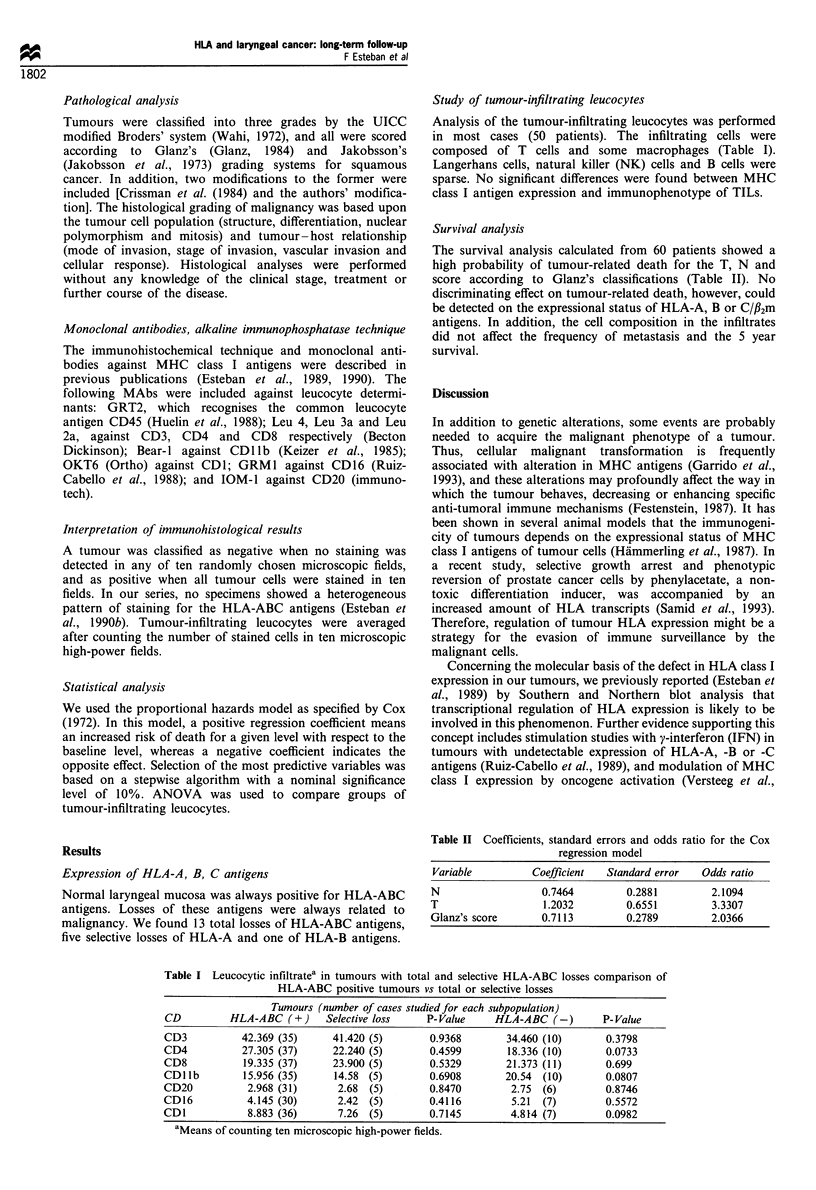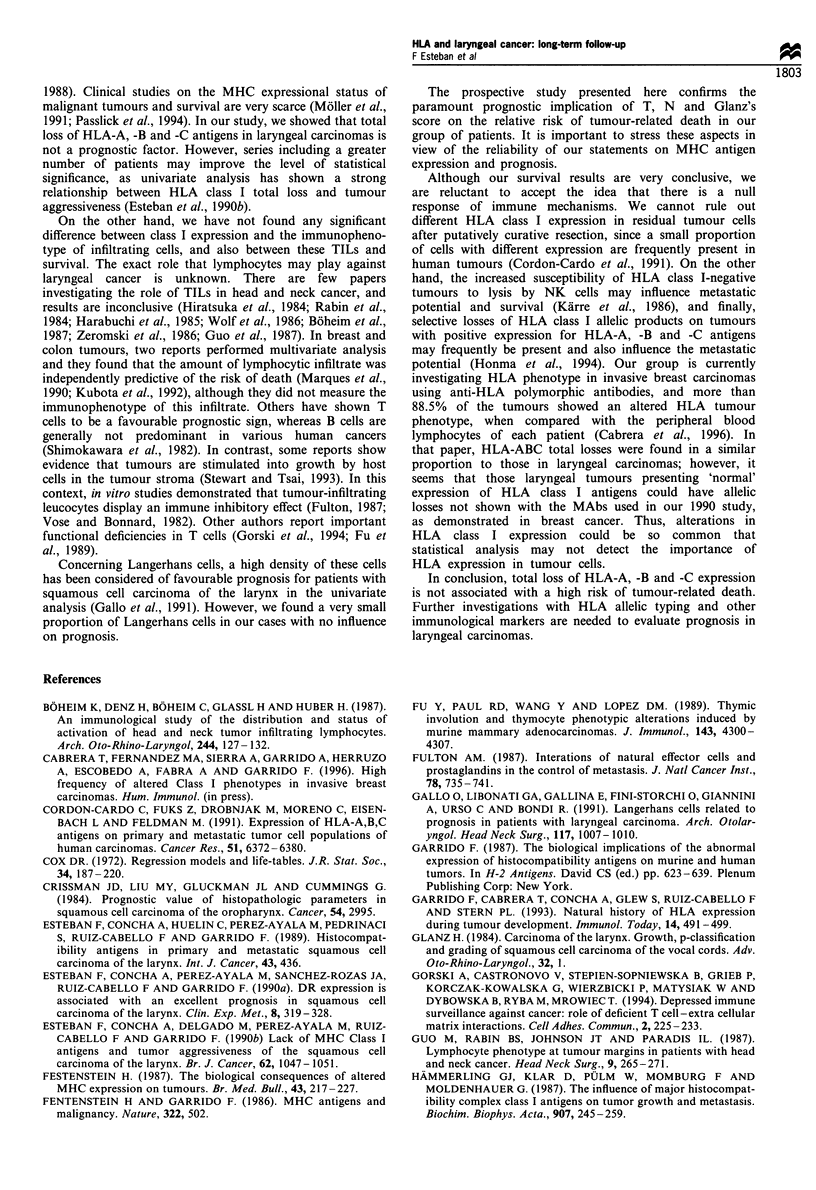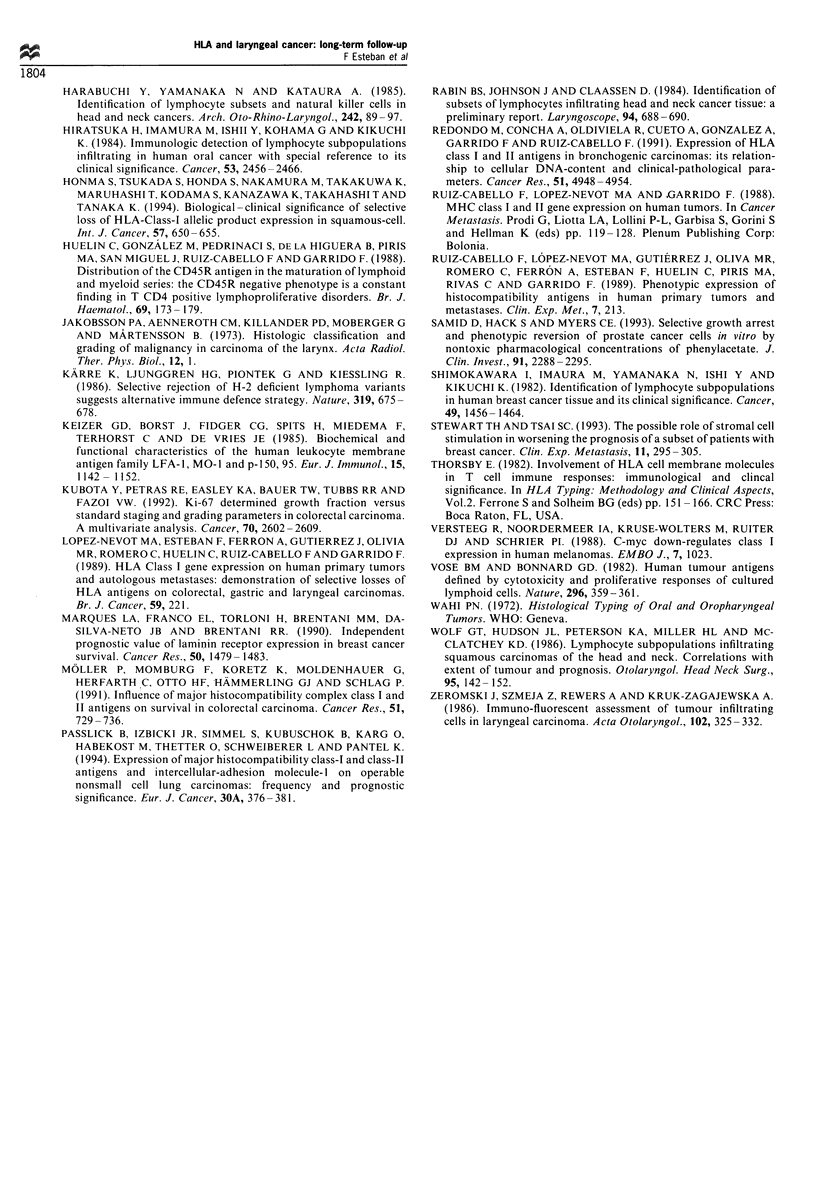# MHC class I antigens and tumour-infiltrating leucocytes in laryngeal cancer: long-term follow-up.

**DOI:** 10.1038/bjc.1996.633

**Published:** 1996-12

**Authors:** F. Esteban, M. Redondo, M. Delgado, F. Garrido, F. Ruiz-Cabello

**Affiliations:** Servicio de Otorrinolaringologia, Hospital Universitario Virgen de Valme, Seville, Spain.

## Abstract

Alteration in MHC class I expression may be used by cancer cells to avoid immune destruction. Much experimental evidence supports this idea, although survival studies are very scarce. To investigate whether the presence or absence of HLA-A, -B and -C antigens in laryngeal carcinoma influences survival, a series of 60 primary laryngeal tumours treated surgically and normal tissues were evaluated in frozen sections for the expression of MHC class I antigens and tumour-infiltrating leucocytes (CD3, CD4, CD8, CD11b, CD1, CD20 and CD16), using monoclonal antibodies and the APAAP, technique. Long-term follow-up from the patients is available, ranging from 6 to 10 years. Thirteen tumours presented total HLA-ABC loss, five selective losses of HLA-A antigens and one absence of HLA-B antigens. Total losses were statistically associated with several clinical and pathological parameters, but there were no differences regarding tumour-infiltrating leucocytes. After conducting a prospective study, only T and N staging and scoring according to Glanz's malignancy classification were found to be independently related to patients' outcome. From our data, we conclude that neither complete loss of HLA class I antigens nor tumour-infiltrating leucocytes appear to influence survival in squamous cell carcinoma of the larynx.


British Journal of Cancer (1996) 74, 1801-1804
© 1996 Stockton Press All rights reserved 0007-0920/96 $12.00

### MHC class I antigens and tumour-infiltrating leucocytes in laryngeal cancer: long-term follow-up

F Esteban[1], M Redondo[2], M Delgado[3], F Garrido[4] and F Ruiz-Cabello[4]

[1]*Servicio de Otorrinolaringología, Hospital Universitario 'Virgen de Valme', Seville;* [2]*Servicio de Análisis Clínicos, Hospital Costa del Sol, Marbella, Málaga;* [3]*Cátedra de Medicina Preventiva, Universidad de Cantabria, Santander;* [4]*Servicio de Analisis Clínicos e Inmunología, Hospital Universitario 'Virgen de las Nieves', Granada, Spain.*

**Summary** Alteration in MHC class I expression may be used by cancer cells to avoid immune destruction. Much experimental evidence supports this idea, although survival studies are very scarce. To investigate whether the presence or absence of HLA-A, -B and -C antigens in laryngeal carcinoma influences survival, a series of 60 primary laryngeal tumours treated surgically and normal tissues were evaluated in frozen sections for the expression of MHC class I antigens and tumour-infiltrating leucocytes (CD3, CD4, CD8, CD11b, CD1, CD20 and CD16), using monoclonal antibodies and the APAAP technique. Long-term follow-up from the patients is available, ranging from 6 to 10 years. Thirteen tumours presented total HLA-ABC loss, five selective losses of HLA-A antigens and one absence of HLA-B antigens. Total losses were statistically associated with several clinical and pathological parameters, but there were no differences regarding tumour-infiltrating leucocytes. After conducting a prospective study, only T and N staging and scoring according to Glanz's malignancy classification were found to be independently related to patients' outcome. From our data, we conclude that neither complete loss of HLA class I antigens nor tumour-infiltrating leucocytes appear to influence survival in squamous cell carcinoma of the larynx.

**Keywords:** laryngeal carcinoma; MHC antigens; tumour-infiltrating leucocytes

MHC class I antigens are membrane glycoproteins involved in effective killing by cytotoxic T cells, which require HLA class I compatibility between T cells and target cells (Thorsby, 1982; Festenstein and Garrido, 1986). Alterations in HLA-ABC expression may be one method used by cancer cells to avoid immune destruction (Garrido, 1987). Decreased HLA expression has been discovered in several human tumours, including breast, lung, skin, colorectal, gastric and laryngeal carcinomas (López-Nevot *et al.*, 1986, 1989; Redondo *et al.*, 1991; Ruiz-Cabello *et al.*, 1989). However, few studies have analysed the mechanisms responsible for such alterations. In previous papers, we investigated changes in the expression of HLA class I antigens during malignant transformation of the laryngeal epithelium and some mechanisms involved (Esteban *et al.*, 1989), and we found several clinical and pathological correlations between HLA-ABC losses and aggressiveness (Esteban *et al.*, 1990*a*). MHC class II antigens were only found in verrucous cell carcinomas, thus DR expression seems to be associated with tumours with excellent prognosis (Esteban *et al.*, 1990*b*). On the other hand, laryngeal tumours are often characterised by an inflammatory reaction that follows the growth of neoplastic elements, but its prognostic value and its functional significance remain to be determined. The aim of the present work is to present the long-term results of the series and to evaluate the prognostic significance of MHC class I antigens and tumour-infiltrating leucocytes (TILs) in patients with laryngeal carcinoma after long-term follow-up.

### Materials and methods

### *Patients*

Sixty patients with squamous cell carcinoma of the larynx were included in the study. None of them received radiotherapy or chemotherapy before surgery. All were male. Age ranged from 44 to 75 years (average 58.68 years). Tumours were classified as originating from the supraglottic region (33,55%), glottis (18,30%), subglottis (2,3.3%) and pyriform sinus (3,5%). Four cases were considered transglottic (6.7%). Thus, there were 57 laryngeal and three hypopharyngeal tumours in the study. Surgical techniques consisted of cordectomy (four cases), frontolateral laryngectomy with epiglottoplasty as popularised by Harvey Tucker (two cases), supraglottic horizontal laryngectomy (12 cases) and total laryngectomy (42 cases). Neck dissection was performed at clinical stage IV disease and at stage III as indicated by the presence of or high risk of cervical metastasis and by the specific site of the primary tumour. A total of 22 patients underwent ipsilateral and 18 bilateral functional neck dissections. Radical ipsilateral neck dissection was done in three patients, and a bilateral staged procedure in four. Post-operative radiation therapy was decided on an individual basis by a multispeciality joint tumour board (otolaryngologist, oncologist and pathologist), and radiotherapy was chosen for 29 patients. Basically, radiotherapy was chosen for all T4 and/or node-positive tumours, since functional neck dissection allows pathological staging for the neck. There were four T1, four T2, 37 T3 and 15 T4 carcinomas. Forty-two patients were classified as NO, six N1, eight N2 and four N3. Follow-up ranged from 66 to 112 months, available for all patients. At present, 19 patients have died: 12 patients as a result of the disease (five neck relapses, two pharyngeal recurrences, three cases with pulmonary metastasis, one case with neck relapse and lung metastasis, one stomal recurrence) and there were seven deaths unrelated to the laryngeal disease (one myocardial infarction; 1 car crash; 1 chronic pulmonary obstructive disease; one patient committed suicide, and three died as a result of other tumours: lung, prostate and oesophagus).

Location and diameter of the primary tumour, T and N pathological staging (according to UICC, 1992 version), number of lymph nodes from the neck dissection and number of lymph node metastases, pathological global staging, blood group and Rh of the patient, age, smoking and alcohol consumption, first symptom–diagnosis intervals and follow-up (in months) were recorded for each case.

Correspondence: F Esteban, Servicio de Otorrinolaringologia, Hospital Universitario 'Virgen de Valme', Ctra. de Cadiz s/n, Seville 41014, Spain
Received 5 March 1996; revised 17 May 1996; accepted 28 June 1996

*Pathological analysis*

Tumours were classified into three grades by the UICC modified Broders' system (Wahi, 1972), and all were scored according to Glanz's (Glanz, 1984) and Jakobsson's (Jakobsson *et al.*, 1973) grading systems for squamous cancer. In addition, two modifications to the former were included [Crissman *et al.* (1984) and the authors' modification]. The histological grading of malignancy was based upon the tumour cell population (structure, differentiation, nuclear polymorphism and mitosis) and tumour–host relationship (mode of invasion, stage of invasion, vascular invasion and cellular response). Histological analyses were performed without any knowledge of the clinical stage, treatment or further course of the disease.

*Monoclonal antibodies, alkaline immunophosphatase technique*

The immunohistochemical technique and monoclonal antibodies against MHC class I antigens were described in previous publications (Esteban *et al.*, 1989, 1990). The following MAbs were included against leucocyte determinants: GRT2, which recognises the common leucocyte antigen CD45 (Huelin *et al.*, 1988); Leu 4, Leu 3a and Leu 2a, against CD3, CD4 and CD8 respectively (Becton Dickinson); Bear-1 against CD11b (Keizer *et al.*, 1985); OKT6 (Ortho) against CD1; GRM1 against CD16 (Ruiz-Cabello *et al.*, 1988); and IOM-1 against CD20 (immunotech).

*Interpretation of immunohistological results*

A tumour was classified as negative when no staining was detected in any of ten randomly chosen microscopic fields, and as positive when all tumour cells were stained in ten fields. In our series, no specimens showed a heterogeneous pattern of staining for the HLA-ABC antigens (Esteban *et al.*, 1990*b*). Tumour-infiltrating leucocytes were averaged after counting the number of stained cells in ten microscopic high-power fields.

*Statistical analysis*

We used the proportional hazards model as specified by Cox (1972). In this model, a positive regression coefficient means an increased risk of death for a given level with respect to the baseline level, whereas a negative coefficient indicates the opposite effect. Selection of the most predictive variables was based on a stepwise algorithm with a nominal significance level of 10%. ANOVA was used to compare groups of tumour-infiltrating leucocytes.

### Results

*Expression of HLA-A, B, C antigens*

Normal laryngeal mucosa was always positive for HLA-ABC antigens. Losses of these antigens were always related to malignancy. We found 13 total losses of HLA-ABC antigens, five selective losses of HLA-A and one of HLA-B antigens.

*Study of tumour-infiltrating leucocytes*

Analysis of the tumour-infiltrating leucocytes was performed in most cases (50 patients). The infiltrating cells were composed of T cells and some macrophages (Table I). Langerhans cells, natural killer (NK) cells and B cells were sparse. No significant differences were found between MHC class I antigen expression and immunophenotype of TILs.

*Survival analysis*

The survival analysis calculated from 60 patients showed a high probability of tumour-related death for the T, N and score according to Glanz's classifications (Table II). No discriminating effect on tumour-related death, however, could be detected on the expressional status of HLA-A, B or C/$\beta_2$m antigens. In addition, the cell composition in the infiltrates did not affect the frequency of metastasis and the 5 year survival.

### Discussion

In addition to genetic alterations, some events are probably needed to acquire the malignant phenotype of a tumour. Thus, cellular malignant transformation is frequently associated with alteration in MHC antigens (Garrido *et al.*, 1993), and these alterations may profoundly affect the way in which the tumour behaves, decreasing or enhancing specific anti-tumoral immune mechanisms (Festenstein, 1987). It has been shown in several animal models that the immunogenicity of tumours depends on the expressional status of MHC class I antigens of tumour cells (Hämmerling *et al.*, 1987). In a recent study, selective growth arrest and phenotypic reversion of prostate cancer cells by phenylacetate, a non-toxic differentiation inducer, was accompanied by an increased amount of HLA transcripts (Samid *et al.*, 1993). Therefore, regulation of tumour HLA expression might be a strategy for the evasion of immune surveillance by the malignant cells.

Concerning the molecular basis of the defect in HLA class I expression in our tumours, we previously reported (Esteban *et al.*, 1989) by Southern and Northern blot analysis that transcriptional regulation of HLA expression is likely to be involved in this phenomenon. Further evidence supporting this concept includes stimulation studies with $\gamma$-interferon (IFN) in tumours with undetectable expression of HLA-A, -B or -C antigens (Ruiz-Cabello *et al.*, 1989), and modulation of MHC class I expression by oncogene activation (Versteeg *et al.*,

**Table II** Coefficients, standard errors and odds ratio for the Cox regression model

| *Variable* | *Coefficient* | *Standard error* | *Odds ratio* |
|---|---|---|---|
| N | 0.7464 | 0.2881 | 2.1094 |
| T | 1.2032 | 0.6551 | 3.3307 |
| Glanz's score | 0.7113 | 0.2789 | 2.0366 |

**Table I** Leucocytic infiltrate[a] in tumours with total and selective HLA-ABC losses comparison of HLA-ABC positive tumours *vs* total or selective losses

| | Tumours (number of cases studied for each subpopulation) | | | | |
|---|---|---|---|---|---|
| *CD* | *HLA-ABC (+)* | *Selective loss* | P-*Value* | *HLA-ABC (−)* | P-*Value* |
| CD3 | 42.369 (35) | 41.420 (5) | 0.9368 | 34.460 (10) | 0.3798 |
| CD4 | 27.305 (37) | 22.240 (5) | 0.4599 | 18.336 (10) | 0.0733 |
| CD8 | 19.335 (37) | 23.900 (5) | 0.5329 | 21.373 (11) | 0.699 |
| CD11b | 15.956 (35) | 14.58 (5) | 0.6908 | 20.54 (10) | 0.0807 |
| CD20 | 2.968 (31) | 2.68 (5) | 0.8470 | 2.75 (6) | 0.8746 |
| CD16 | 4.145 (30) | 2.42 (5) | 0.4116 | 5.21 (7) | 0.5572 |
| CD1 | 8.883 (36) | 7.26 (5) | 0.7145 | 4.814 (7) | 0.0982 |

[a]Means of counting ten microscopic high-power fields.

1988). Clinical studies on the MHC expressional status of malignant tumours and survival are very scarce (Möller *et al.*, 1991; Passlick *et al.*, 1994). In our study, we showed that total loss of HLA-A, -B and -C antigens in laryngeal carcinomas is not a prognostic factor. However, series including a greater number of patients may improve the level of statistical significance, as univariate analysis has shown a strong relationship between HLA class I total loss and tumour aggressiveness (Esteban *et al.*, 1990*b*).

On the other hand, we have not found any significant difference between class I expression and the immunophenotype of infiltrating cells, and also between these TILs and survival. The exact role that lymphocytes may play against laryngeal cancer is unknown. There are few papers investigating the role of TILs in head and neck cancer, and results are inconclusive (Hiratsuka *et al.*, 1984; Rabin *et al.*, 1984; Harabuchi *et al.*, 1985; Wolf *et al.*, 1986; Böheim *et al.*, 1987; Zeromski *et al.*, 1986; Guo *et al.*, 1987). In breast and colon tumours, two reports performed multivariate analysis and they found that the amount of lymphocytic infiltrate was independently predictive of the risk of death (Marques *et al.*, 1990; Kubota *et al.*, 1992), although they did not measure the immunophenotype of this infiltrate. Others have shown T cells to be a favourable prognostic sign, whereas B cells are generally not predominant in various human cancers (Shimokawara *et al.*, 1982). In contrast, some reports show evidence that tumours are stimulated into growth by host cells in the tumour stroma (Stewart and Tsai, 1993). In this context, *in vitro* studies demonstrated that tumour-infiltrating leucocytes display an immune inhibitory effect (Fulton, 1987; Vose and Bonnard, 1982). Other authors report important functional deficiencies in T cells (Gorski *et al.*, 1994; Fu *et al.*, 1989).

Concerning Langerhans cells, a high density of these cells has been considered of favourable prognosis for patients with squamous cell carcinoma of the larynx in the univariate analysis (Gallo *et al.*, 1991). However, we found a very small proportion of Langerhans cells in our cases with no influence on prognosis.

The prospective study presented here confirms the paramount prognostic implication of T, N and Glanz's score on the relative risk of tumour-related death in our group of patients. It is important to stress these aspects in view of the reliability of our statements on MHC antigen expression and prognosis.

Although our survival results are very conclusive, we are reluctant to accept the idea that there is a null response of immune mechanisms. We cannot rule out different HLA class I expression in residual tumour cells after putatively curative resection, since a small proportion of cells with different expression are frequently present in human tumours (Cordon-Cardo *et al.*, 1991). On the other hand, the increased susceptibility of HLA class I-negative tumours to lysis by NK cells may influence metastatic potential and survival (Kärre *et al.*, 1986), and finally, selective losses of HLA class I allelic products on tumours with positive expression for HLA-A, -B and -C antigens may frequently be present and also influence the metastatic potential (Honma *et al.*, 1994). Our group is currently investigating HLA phenotype in invasive breast carcinomas using anti-HLA polymorphic antibodies, and more than 88.5% of the tumours showed an altered HLA tumour phenotype, when compared with the peripheral blood lymphocytes of each patient (Cabrera *et al.*, 1996). In that paper, HLA-ABC total losses were found in a similar proportion to those in laryngeal carcinomas; however, it seems that those laryngeal tumours presenting 'normal' expression of HLA class I antigens could have allelic losses not shown with the MAbs used in our 1990 study, as demonstrated in breast cancer. Thus, alterations in HLA class I expression could be so common that statistical analysis may not detect the importance of HLA expression in tumour cells.

In conclusion, total loss of HLA-A, -B and -C expression is not associated with a high risk of tumour-related death. Further investigations with HLA allelic typing and other immunological markers are needed to evaluate prognosis in laryngeal carcinomas.

### References

BÖHEIM K, DENZ H, BÖHEIM C, GLASSL H AND HUBER H. (1987). An immunological study of the distribution and status of activation of head and neck tumor infiltrating lymphocytes. *Arch. Oto-Rhino-Laryngol*, **244**, 127–132.

CABRERA T, FERNANDEZ MA, SIERRA A, GARRIDO A, HERRUZO A, ESCOBEDO A, FABRA A AND GARRIDO F. (1996). High frequency of altered Class I phenotypes in invasive breast carcinomas. *Hum. Immunol.* (in press).

CORDON-CARDO C, FUKS Z, DROBNJAK M, MORENO C, EISENBACH L AND FELDMAN M. (1991). Expression of HLA-A,B,C antigens on primary and metastatic tumor cell populations of human carcinomas. *Cancer Res.*, **51**, 6372–6380.

COX DR. (1972). Regression models and life-tables. *J.R. Stat. Soc.*, **34**, 187–220.

CRISSMAN JD, LIU MY, GLUCKMAN JL AND CUMMINGS G. (1984). Prognostic value of histopathologic parameters in squamous cell carcinoma of the oropharynx. *Cancer*, **54**, 2995.

ESTEBAN F, CONCHA A, HUELIN C, PEREZ-AYALA M, PEDRINACI S, RUIZ-CABELLO F AND GARRIDO F. (1989). Histocompatibility antigens in primary and metastatic squamous cell carcinoma of the larynx. *Int. J. Cancer*, **43**, 436.

ESTEBAN F, CONCHA A, PEREZ-AYALA M, SANCHEZ-ROZAS JA, RUIZ-CABELLO F AND GARRIDO F. (1990*a*). DR expression is associated with an excellent prognosis in squamous cell carcinoma of the larynx. *Clin. Exp. Met.*, **8**, 319–328.

ESTEBAN F, CONCHA A, DELGADO M, PEREZ-AYALA M, RUIZ-CABELLO F AND GARRIDO F. (1990*b*) Lack of MHC Class I antigens and tumor aggressiveness of the squamous cell carcinoma of the larynx. *Br. J. Cancer*, **62**, 1047–1051.

FESTENSTEIN H. (1987). The biological consequences of altered MHC expression on tumours. *Br. Med. Bull.*, **43**, 217–227.

FENTENSTEIN H AND GARRIDO F. (1986). MHC antigens and malignancy. *Nature*, **322**, 502.

FU Y, PAUL RD, WANG Y AND LOPEZ DM. (1989). Thymic involution and thymocyte phenotypic alterations induced by murine mammary adenocarcinomas. *J. Immunol.*, **143**, 4300–4307.

FULTON AM. (1987). Interations of natural effector cells and prostaglandins in the control of metastasis. *J. Natl Cancer Inst.*, **78**, 735–741.

GALLO O, LIBONATI GA, GALLINA E, FINI-STORCHI O, GIANNINI A, URSO C AND BONDI R. (1991). Langerhans cells related to prognosis in patients with laryngeal carcinoma. *Arch. Otolaryngol. Head Neck Surg.*, **117**, 1007–1010.

GARRIDO F. (1987). The biological implications of the abnormal expression of histocompatibility antigens on murine and human tumors. In *H-2 Antigens*. David CS (ed.) pp. 623–639. Plenum Publishing Corp: New York.

GARRIDO F, CABRERA T, CONCHA A, GLEW S, RUIZ-CABELLO F AND STERN PL. (1993). Natural history of HLA expression during tumour development. *Immunol. Today*, **14**, 491–499.

GLANZ H. (1984). Carcinoma of the larynx. Growth, p-classification and grading of squamous cell carcinoma of the vocal cords. *Adv. Oto-Rhino-Laryngol.*, **32**, 1.

GORSKI A, CASTRONOVO V, STEPIEN-SOPNIEWSKA B, GRIEB P, KORCZAK-KOWALSKA G, WIERZBICKI P, MATYSIAK W AND DYBOWSKA B, RYBA M, MROWIEC T. (1994). Depressed immune surveillance against cancer: role of deficient T cell–extra cellular matrix interactions. *Cell Adhes. Commun.*, **2**, 225–233.

GUO M, RABIN BS, JOHNSON JT AND PARADIS IL. (1987). Lymphocyte phenotype at tumour margins in patients with head and neck cancer. *Head Neck Surg.*, **9**, 265–271.

HÄMMERLING GJ, KLAR D, PÜLM W, MOMBURG F AND MOLDENHAUER G. (1987). The influence of major histocompatibility complex class I antigens on tumor growth and metastasis. *Biochim. Biophys. Acta.*, **907**, 245–259.

HARABUCHI Y, YAMANAKA N AND KATAURA A. (1985). Identification of lymphocyte subsets and natural killer cells in head and neck cancers. *Arch. Oto-Rhino-Laryngol.*, **242,** 89–97.

HIRATSUKA H, IMAMURA M, ISHII Y, KOHAMA G AND KIKUCHI K. (1984). Immunologic detection of lymphocyte subpopulations infiltrating in human oral cancer with special reference to its clinical significance. *Cancer*, **53,** 2456–2466.

HONMA S, TSUKADA S, HONDA S, NAKAMURA M, TAKAKUWA K, MARUHASHI T, KODAMA S, KANAZAWA K, TAKAHASHI T AND TANAKA K. (1994). Biological–clinical significance of selective loss of HLA-Class-I allelic product expression in squamous-cell. *Int. J. Cancer*, **57,** 650–655.

HUELIN C, GONZÁLEZ M, PEDRINACI S, DE LA HIGUERA B, PIRIS MA, SAN MIGUEL J, RUIZ-CABELLO F AND GARRIDO F. (1988). Distribution of the CD45R antigen in the maturation of lymphoid and myeloid series: the CD45R negative phenotype is a constant finding in T CD4 positive lymphoproliferative disorders. *Br. J. Haematol.*, **69,** 173–179.

JAKOBSSON PA, AENNEROTH CM, KILLANDER PD, MOBERGER G AND MÅRTENSSON B. (1973). Histologic classification and grading of malignancy in carcinoma of the larynx. *Acta Radiol. Ther. Phys. Biol.*, **12,** 1.

KÄRRE K, LJUNGGREN HG, PIONTEK G AND KIESSLING R. (1986). Selective rejection of H-2 deficient lymphoma variants suggests alternative immune defence strategy. *Nature*, **319,** 675–678.

KEIZER GD, BORST J, FIDGER CG, SPITS H, MIEDEMA F, TERHORST C AND DE VRIES JE (1985). Biochemical and functional characteristics of the human leukocyte membrane antigen family LFA-1, MO-1 and p-150, 95. *Eur. J. Immunol.*, **15,** 1142 – 1152.

KUBOTA Y, PETRAS RE, EASLEY KA, BAUER TW, TUBBS RR AND FAZOI VW. (1992). Ki-67 determined growth fraction versus standard staging and grading parameters in colorectal carcinoma. A multivariate analysis. *Cancer*, **70,** 2602–2609.

LOPEZ-NEVOT MA, ESTEBAN F, FERRON A, GUTIERREZ J, OLIVIA MR, ROMERO C, HUELIN C, RUIZ-CABELLO F AND GARRIDO F. (1989). HLA Class I gene expression on human primary tumors and autologous metastases: demonstration of selective losses of HLA antigens on colorectal, gastric and laryngeal carcinomas. *Br. J. Cancer*, **59,** 221.

MARQUES LA, FRANCO EL, TORLONI H, BRENTANI MM, DA-SILVA-NETO JB AND BRENTANI RR. (1990). Independent prognostic value of laminin receptor expression in breast cancer survival. *Cancer Res.*, **50,** 1479–1483.

MÖLLER P, MOMBURG F, KORETZ K, MOLDENHAUER G, HERFARTH C, OTTO HF, HÄMMERLING GJ AND SCHLAG P. (1991). Influence of major histocompatibility complex class I and II antigens on survival in colorectal carcinoma. *Cancer Res.*, **51,** 729–736.

PASSLICK B, IZBICKI JR, SIMMEL S, KUBUSCHOK B, KARG O, HABEKOST M, THETTER O, SCHWEIBERER L AND PANTEL K. (1994). Expression of major histocompatibility class-I and class-II antigens and intercellular-adhesion molecule-1 on operable nonsmall cell lung carcinomas: frequency and prognostic significance. *Eur. J. Cancer*, **30A,** 376–381.

RABIN BS, JOHNSON J AND CLAASSEN D. (1984). Identification of subsets of lymphocytes infiltrating head and neck cancer tissue: a preliminary report. *Laryngoscope*, **94,** 688–690.

REDONDO M, CONCHA A, OLDIVIELA R, CUETO A, GONZALEZ A, GARRIDO F AND RUIZ-CABELLO F. (1991). Expression of HLA class I and II antigens in bronchogenic carcinomas: its relationship to cellular DNA-content and clinical-pathological parameters. *Cancer Res.*, **51,** 4948–4954.

RUIZ-CABELLO F, LOPEZ-NEVOT MA AND GARRIDO F. (1988). MHC class I and II gene expression on human tumors. In *Cancer Metastasis.* Prodi G, Liotta LA, Lollini P-L, Garbisa S, Gorini S and Hellman K (eds) pp. 119–128. Plenum Publishing Corp: Bolonia.

RUIZ-CABELLO F, LÓPEZ-NEVOT MA, GUTIÉRREZ J, OLIVA MR, ROMERO C, FERRÓN A, ESTEBAN F, HUELIN C, PIRIS MA, RIVAS C AND GARRIDO F. (1989). Phenotypic expression of histocompatibility antigens in human primary tumors and metastases. *Clin. Exp. Met.*, **7,** 213.

SAMID D, HACK S AND MYERS CE. (1993). Selective growth arrest and phenotypic reversion of prostate cancer cells *in vitro* by nontoxic pharmacological concentrations of phenylacetate. *J. Clin. Invest.*, **91,** 2288–2295.

SHIMOKAWARA I, IMAURA M, YAMANAKA N, ISHI Y AND KIKUCHI K. (1982). Identification of lymphocyte subpopulations in human breast cancer tissue and its clinical significance. *Cancer*, **49,** 1456–1464.

STEWART TH AND TSAI SC. (1993). The possible role of stromal cell stimulation in worsening the prognosis of a subset of patients with breast cancer. *Clin. Exp. Metastasis*, **11,** 295–305.

THORSBY E. (1982). Involvement of HLA cell membrane molecules in T cell immune responses: immunological and clincal significance. In *HLA Typing: Methodology and Clinical Aspects*, Vol.2. Ferrone S and Solheim BG (eds) pp. 151–166. CRC Press: Boca Raton, FL, USA.

VERSTEEG R, NOORDERMEER IA, KRUSE-WOLTERS M, RUITER DJ AND SCHRIER PI. (1988). C-myc down-regulates class I expression in human melanomas. *EMBO J.*, **7,** 1023.

VOSE BM AND BONNARD GD. (1982). Human tumour antigens defined by cytotoxicity and proliferative responses of cultured lymphoid cells. *Nature*, **296,** 359–361.

WAHI PN. (1972). *Histological Typing of Oral and Oropharyngeal Tumors.* WHO: Geneva.

WOLF GT, HUDSON JL, PETERSON KA, MILLER HL AND MC-CLATCHEY KD. (1986). Lymphocyte subpopulations infiltrating squamous carcinomas of the head and neck. Correlations with extent of tumour and prognosis. *Otolaryngol. Head Neck Surg.*, **95,** 142–152.

ZEROMSKI J, SZMEJA Z, REWERS A AND KRUK-ZAGAJEWSKA A. (1986). Immuno-fluorescent assessment of tumour infiltrating cells in laryngeal carcinoma. *Acta Otolaryngol.*, **102,** 325–332.